# Improving the Catalytic Activity and Thermostability of FAST-PETase with a Multifunctional Short Peptide

**DOI:** 10.3390/biom15060888

**Published:** 2025-06-18

**Authors:** Jun Yang, Binyang Deng, Pingan Liao, Siyu Lin, Liqi Zheng, Xing Yang, Fei Wang, Chao Zhai, Lixin Ma

**Affiliations:** State Key Laboratory of Biocatalysis and Enzyme Engineering, Hubei Key Laboratory of Industrial Biotechnology, School of Life Sciences, Hubei University, Wuhan 430062, China; youcojun@stu.hubu.edu.cn (J.Y.); 202311107010110@stu.hubu.edu.cn (B.D.); 202311107010114@stu.hubu.edu.cn (P.L.); 201811110711009@stu.hubu.edu.cn (S.L.); 202421107011635@stu.hubu.edu.cn (L.Z.); 202421107011886@stu.hubu.edu.cn (X.Y.); wangfei@hubu.edu.cn (F.W.)

**Keywords:** PETase, multifunctional short peptide, thermostability, catalytic activity

## Abstract

Previous reports indicated that self-assembling amphipathic peptide S1v1 (AEAEAHAH)_2_ significantly enhances the soluble expression, thermostability, and activity of the target proteins when fused to them. In order to obtain high-efficiency enzymes for the large-scale degradation of polyethylene terephthalate (PET), this multifunctional peptide was fused to the N- and C-terminus of FAST-PETase, a variant of *Ideonella sakaiensis* PETase (*Is*PETase), with a PT-linker (TTVTTPQTS) harbored between the target protein and the multifunctional peptide. Consistent with previous reports, S1v1 increased the solubility of FAST-PETase slightly. Moreover, it increased the activity of FAST-PETase dramatically. The amount of terephthalic acid (TPA) and mono(2-hydroxyethyl) terephthalic acid (MHET) released from PET substrate after 24 h of digestion at 50°C by fusion enzymes bearing N- and C-terminal S1v1 tag was approximately 2.9- and 4.6-fold that of FAST-PETase, respectively. Furthermore, the optimal temperature and thermostability of the fusion proteins increased in comparison with FAST-PETase. The present study provides a novel strategy to improve the depolymerization efficiency of FAST-PETase.

## 1. Introduction

Synthesized plastics are widely used in textile, packaging, and bottle producing industries due to their high durability, elasticity, strength, and resistance to chemicals [1]. On the other hand, the same characteristics caused serious problems to their natural degradation [1,2]. Efficient, low-cost, and eco-friendly methods for the degradation of synthetic plastics are in urgent need considering the increasing consumption and accommodation of plastics in the environment. In 2005, Muller et al. reported the biodegradation of PET with a PETase-like enzyme secreted by *Thermobifida fusca* (*Tf*Cut) for the first time [3]. Since then, many PET hydrolyzing enzymes were mined and applied to the depolymerization of PET. PET is by far the most studied artificial polymer in terms of biodegradation [4]. In 2016, the first PETase was identified from *Ideonella sakaiensis* 201-F6 and named as *Is*PETase [5]. Although this enzyme demonstrated high activity to PET, it is thermosensitive and loses most of its activity within 24 h at 37°C. Since PET has a glass transition temperature (*Tg*) of 76°C and its depolymerization tends to happen at high temperature, *Is*PETase is unsuitable for industrial applications. After the structure and the catalytic mechanism of *Is*PETase were clarified [6,7], intensive studies were carried out to improve its activity and thermostability. Several outstanding variants were generated, including ThermoPETase [8], DuraPETase [9], FAST-PETase [10], and Hot-PETase [11], etc. FAST-PETase was generated in 2022 through computational learning. It is capable of depolymerizing untreated, postconsumer-PET from 51 PET products in just 1 week. This enzyme demonstrated great potential in the green cycling of PET plastics.

However, the high-level expression of *Is*PETase and its variants, with *Escherichia coli* as the host, remains a challenge to date. Native *Is*PETase is secreted extracellularly by *Ideonella sakaiensis* [5]. Therefore, the secretion expression of *Is*PETase and variants with *E. coli* and *Bacillus subtilis* has been investigated using different signal sequences and promoters. However, the titers of the target protein were relatively low [12,13,14,15]. Meanwhile, its intracellular expression with *E. coli* bearing an enhanced disulfide bond formation ability was investigated. With an optimized medium, the titer of *Is*PETase and its variants reached approximately 1 mg/L [16]. Our previous work indicated that partially glycosylated *Is*PETase and FAST-PETase, prepared with a combination of *Pichia pastoris* heterologous expression system and endo-β-N-acetylglucosaminidase H (Endo H) treatment, demonstrated high yield and elevated enzymatic activity in comparison with the recombinant enzymes expressed with *E. coli* [17]. In addition, the soluble expression and the titer of *Is*PETase were improved when carbohydrate-binding module 66 (CBM66) was fused to the enzyme [18]. These results implied the modification of *Is*PETase itself maybe another efficient approach to improve the yield and activity of the enzyme besides of the optimization of the expression systems. Self-assembling amphipathic peptides (SAPs) are a class of multifunctional peptides composed of alternative hydrophilic and hydrophobic residues and can spontaneously aggregate into ordered nano-structures in solution [19]. S1, with the sequence of (AEAEAKAK)_2_, is an SAP derived from the Z-DNA-binding protein of *Saccharomyces cerevisiae* [20]. It was applied as a fusion tag to increase the thermostabilities and activities of α-amylase [21] and nitrile hydratase [22]. Subsequently, the lysine residues in S1 were replaced with histidine residues to gain S1v1 with the sequence of (AEAEAHAH)_2_. This artificial peptide fused to polygalacturonate lyase (PGL), lipoxygenase (LOX), green fluorescent protein (GFP) [23], and ZEN lactone hydrolase [24] with a PT-linker (PTPPTTPTPPTTPTPTP) among the SAP and target protein. The results indicated S1v1 was able to enhance the expression, purification, thermostability, and activity of the target proteins significantly. In the present study, the S1v1 multifunctional peptide was fused to the N- and C-terminus of FAST-PETase with a PT-linker in the middle. Consistent with previous reports, S1v1 increased the solubility of the FAST-PETase in *E. coli* BL21-CondonPlus (DE3)-RIPL. Moreover, it increased the activity and thermostability of FAST-PETase dramatically.

## 2. Materials and Methods

### 2.1. Bacteria, Plasmids, Media, and Reagents

For gene cloning, *E. coli* DH5α was stored in our lab. *E. coli* RosettaBlue (DE3), BL21-CondonPlus (DE3)-RIPL, and Origami 2 (DE3) were purchased from TransGen Biotechnology (Beijing, China). Plasmid pET28a was stored in our lab. Luria–Bertani (LB) media were prepared as described in the Manual of Molecular Cloning [25]. A PET film was purchased from Goodfellow Ltd. (ES301445, Huntingdon, UK). The TPA was purchased from Sigma-Aldrich (St. Louis, MO, USA). MHET was purchased from Aladdin (Shanghai, China). *p*-NPB was purchased from Coolaber (Beijing, China). All other chemicals were analytical reagents.

### 2.2. Construction of Plasmids for the Expression of FAST-PETase Fused with or without S1v1 Tag

The ORF encoding FAST-PETase was synthesized by Sangon (Shanghai, China) and cloned into a pET28a vector through the TLTC method [26] to generate the expression vector pET28a-FAST-PETase. To facilitate the purification, a 6×His tag was fused to the C-terminus of FAST-PETase. The recombinant plasmid was verified by Sanger sequencing.

Next, the coding sequence of the multifunctional tag was synthesized and inserted into pET28a-FAST-PETase through TLTC to generate pET28a-S1v1-FAST-PETase and pET28a-FAST-PETase-S1v1. A 6×His tag was fused to the C-terminus of the fusion proteins, and a PT-linker (TTVTTPQTS) was harbored between the target protein and S1v1 tag. The recombinant plasmids were verified by Sanger sequencing.

### 2.3. Expression of FAST-PETase Fused with or without S1v1 Tag

To induce the expression of the target genes, the recombinant plasmids were transformed into *E. coli* RosettaBlue (DE3), BL21-CondonPlus (DE3)-RIPL, and Origami 2 (DE3). The transformants were cultivated at 37°C with continuous shaking until OD_600_ reached 0.6–0.8. Protein expression was then induced by adding 0.5 mM Isopropyl-β-D-thiogalactopyranoside (IPTG) at 18°C for 18 h. The cultures were centrifuged at 8000 rpm for 10 min, the supernatant was discarded, and the cell pellet was stored at −80°C for subsequent analysis.

### 2.4. Purification of the Recombinant Proteins with Ni-NTA Affinity Chromatography

Cells were collected and resuspended in lysis buffer (50 mM Tris-HCl; 200 mM NaCl; 50 mM NaH_2_PO_4_; 10 mM Imidazole; 5% Glycerol, pH 8.0) with lysozyme at a final concentration of 1 mg/mL. Samples were ultrasonicated to break the cells. The crude cell lysate was then centrifugated at 13,000 rpm for 10 min, and the supernatant was applied to Ni-NTA beads for affinity purification. The column was washed twice with 2 column volumes of wash buffer (50 mM Tris-HCl; 200 mM NaCl; 50 mM NaH_2_PO_4_; 30 mM Imidazole, pH 8.0). One column volume of elution buffer (50 mM Tris-HCl; 200 mM NaCl; 50 mM NaH_2_PO_4_; 300 mM Imidazole, pH 8.0) was used to recover the target protein. The sample was then collected and dialyzed with a Millipore 10 kDa cut-off membrane at 4°C to remove ions and salts, followed by resuspension with storage buffer (50 mM Tris-HCl, 300 mM NaCl, pH 7.5). The obtained proteins were flash-frozen in liquid nitrogen and stored at −80°C for subsequent use.

### 2.5. Sodium Dodecyl Sulfate Polyacrylamide Gel Electrophoresis (SDS-PAGE)

The samples were separated via SDS-PAGE using 12% (*w*/*v*) polyacrylamide gels, followed by staining with Coomassie Brilliant Blue R-250. The protein concentrations were determined using the Bradford kit (Beyotime, Shanghai, China); bovine serum albumin was used as the standard.

### 2.6. Western Blotting

Protein expression was analyzed by western blotting. Proteins were separated on 12% SDS-PAGE gels and transferred to PVDF membranes. The membranes were blocked with 5% non-fat milk and incubated with His-tag primary antibody (mouse monoclonal, ABclonal, Wuhan, China) and HRP-conjugated secondary antibody (Goat anti-Mouse IgG (H+L), ABclonal, Wuhan, China) sequentially. Both antibodies were diluted 1:5000 in 5% non-fat milk. Target proteins were detected using a chemi-luminescent HRP substrate (Millipore Corporation, Billerica, MA, USA) following standard protocols. Original figures can be found in Supplementary Materials.

### 2.7. High-Performance Liquid Chromatography (HPLC) to Analyze the Activity of PETase

To investigate the activity of the recombinant FAST-PETase with or without the multifunctional peptide, the PET films (GfPET, 6 mm in diameter, 8 mg, crystallinity of 7.3%) were incubated with 500 nM purified enzymes in 50 mM Glycine-NaOH buffer (pH 9) at 50°C. After the treatment, the samples were centrifugated at 13,000× *g* for 15 min and analyzed using high-performance liquid chromatography (Shimadzu LC-20AD, Kyoto, Japan) equipped with an InerSustain C18 column (Shimadzu, Kyoto, Japan (4.6 × 250 mM, 5 μm)). The C18 column was eluted using solvent A (20 mM phosphate buffer, pH 2.5) and solvent B (methanol) with a linear gradient from 25% to 85% methanol in 25 min. The mobile-phase flow rate was set at 0.8 mL/min, and the effluent was detected at 240 nm. Concentrations of the PET hydrolysis products MHET and TPA were determined using standards of known concentration. All samples were analyzed in triplicate in each independent experiment, and the average values with standard deviations were calculated.

### 2.8. Kinetic Analysis

The *K_M_* and *k_cat_* values for FAST-PETase and FAST-PETase fused with the S1v1 tag were determined using 0.25 to 2.5 mM of *p*-NPB diluted with 50 mM potassium phosphate buffer (pH 7.5) as the substrate at 50°C or 55°C, as described previously [27,28]. Samples were set up in a clear 96-well plate, with each well containing 200 μL of *p*-NPB and 2.5 μL of enzyme solution (40.5 μM). Negative controls (2.5 μL buffer) were set up for subtraction from the background rate due to non-enzymatic hydrolysis of *p*-NPB. After initial mixing, the absorbance of the samples at a wavelength of 347 nm was measured on a microplate reader (Biotek, Winooski, VT, USA) every 30 s for 6 min [29]. Subtracted initial rates were plotted as a function of *p*-NP concentration and fit to the Michaelis–Menten equation (GraphPad Prism 10).

### 2.9. Thermostability Assay

The thermostability of the recombinant FAST-PETase with or without the S1v1 tag was evaluated by measuring residual *p*-nitrophenyl butyrate (*p*-NPB) activity after incubation at various temperatures. Purified enzymes were incubated at 4°C, 28°C, 37°C, 50°C, and 55°C for 1 h in 50 mM potassium phosphate buffer (pH 7.5). Following incubation, the enzymatic activity of FAST-PETase was measured at 50°C, while the activity of FAST-PETase with the S1v1 tag was measured at 55°C, using *p*-NPB as the substrate. Residual activity was normalized to 100% based on the activity of the enzyme stored at 4°C for 1 h, which served as the reference condition with minimal thermal stress. All measurements were performed in triplicate, and the average values with standard deviations were calculated.

### 2.10. Melting Temperatures Assay with nanoDSF

The Prometheus NT.48 instrument (NanoTemper Technologies, Munich, Germany) was used to determine the melting temperatures. The capillaries were filled with 10 μL samples and placed on the sample holder. A temperature gradient of 1°C·min^−1^ from 20 to 100°C was applied and the intrinsic protein fluorescence was recorded at 330 and 350 nm.

### 2.11. Molecular Docking

The structures of FAST-PETase fused with S1v1 tag were predicted with AlphaFold 3 [30]. The structures of FAST-PETase (PDB ID: 7SH6) and S1v1-tagged FAST-PETase were preprocessed by removing water molecules and adding hydrogen atoms. Similarly, the 3PET structure was prepared by adding hydrogen atoms to study the interaction between S1v1-tagged FAST-PETase (receptor) and 3PET (ligand). A grid box was defined to locate potential binding sites on the protein. Molecular docking was performed using AutoDock Vina 1.1.2, generating multiple ligand conformations. The conformation with the highest score was chosen for further analysis. Interactions between the receptors and the ligand were visualized using Discovery Studio 2019 Client software.

## 3. Results

### 3.1. Expression of FAST-PETase Fused with or without the Multifunctional Peptide

**Figure 1:**
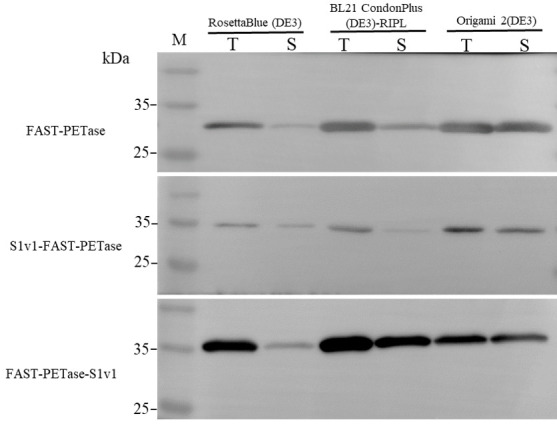
Western blotting analysis of target proteins expression in three *E. coli* strains. M. protein molecular weight marker (size of each band is indicated on left). T stands for total proteins of cell lysate; S stands for supernatant of cell lysate.

The multifunctional short peptide S1v1 was fused to N- or C-terminus of FAST-PETase. A PT-linker was added between the peptide and FAST-PETase. The fusion proteins were named as S1v1-FAST-PETase and FAST-PETase-S1v1. Their sequences were indicated in Supplementary Figures S1 and S2, respectively. FAST-PETase and both fusion proteins were expressed in three *E. coli* expression strains (Figure 1). The results of western blotting indicated that most of the target proteins were insoluble when expressed in *E. coli* RosettaBlue (DE3). The solubility of FAST-PETase-S1v1 was promoted when *E. coli* BL21 CondonPlus (DE3)-RIPL was used as the host while FAST-PETase and S1v1-FAST-PETase remained insoluble. The soluble fraction increased dramatically when the three target proteins were expressed in *E. coli* Origami 2(DE3). Therefore, target proteins expressed in *E. coli* Origami 2(DE3) were used for further study.

### 3.2. The Activities of FAST-PETase Fused with or without the Multifunctional Peptide

**Figure 2:**
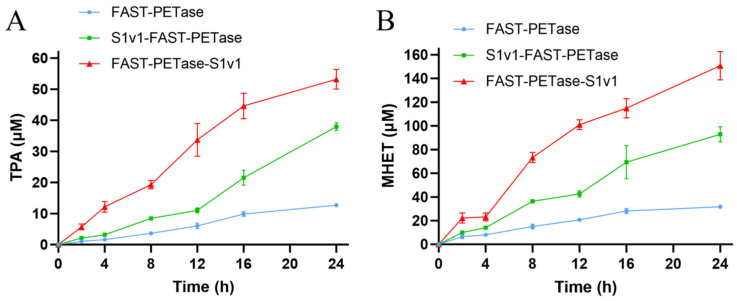
Time course of PET monomers released from GfPET films during digestion with FAST-PETase, FAST-PETase-S1v1, and S1v1-FAST-PETase. (**A**) Time course of TPA released during digestion. (**B**) Time course of MHET released during digestion. Experiments were performed in triplicate, and standard deviations are indicated.

The recombinant FAST-PETase, FAST-PETase-S1v1, and S1v1-FAST-PETase were expressed in *E. coli* and subsequently purified using Ni-NTA resin (Supplementary Figure S3). We analyzed the hydrolytic activity of these enzymes in relation to GfPET. The results of HPLC indicated that the depolymeriziation activity of the fusion proteins was significantly higher than that of FAST-PETase (Figure 2). The amount of TPA released after 24 h of digestion at 50°C with FAST-PETase-S1v1 and S1v1-FAST-PETase was approximately 4.2-fold and 3.0-fold higher than that of FAST-PETase (Figure 2A), and the released MHET was 4.8-fold and 2.9-fold higher than that of FAST-PETase (Figure 2B).

### 3.3. The Thermostability of FAST-PETase Fused with or without S1v1 Tag

**Figure 3:**
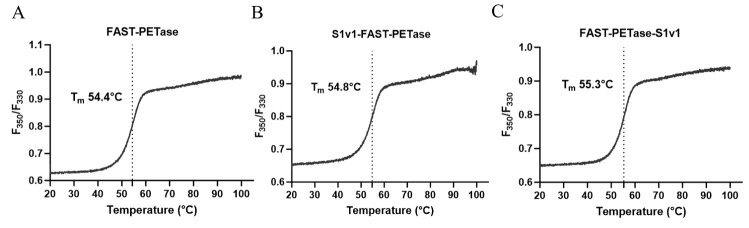
Determination of T_m_ values for FAST-PETase, S1v1-FAST-PETase, and FAST-PETase-S1v1 using nanoDSF.  (**A**) The F350/F330 thermal unfolding curve for FAST-PETase; (**B)** The F350/F330 thermal unfolding curve for S1v1-FAST-PETase; (**C**) The F350/F330 thermal unfolding curve for FAST-PETase-S1v1.

**Figure 4:**
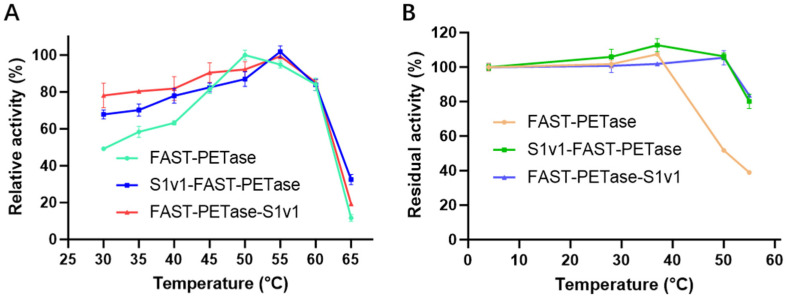
The optimal temperature and thermostability of FAST-PETase with or without S1v1 tag. (**A**) The optimal temperature of FAST-PETase with or without S1v1 tag. (**B**) The thermostability of FAST-PETase with or without S1v1 tag. After incubation at 4°C for 1 h, the remaining activities of FAST-PETase, with or without the fusion tag, were set to 100%. Experiments were performed in triplicate, and standard deviations are indicated.

T_m_ of FAST-PETase, FAST-PETase-S1v1, and S1v1-FAST-PETase were measured with nanoDSF. The results indicated that the T_m_ values of FAST-PETase-S1v1 and S1v1-FAST-PETase were slightly higher than that of FAST-PETase (Figure 3). Consistent with the increased T_m_, FAST-PETase-S1v1 and S1v1-FAST-PETase indicated higher optimal temperature and thermostability. The optimal temperature of FAST-PETase was 50°C, while it was 55°C for FAST-PETase-S1v1 and S1v1-FAST-PETase (Figure 4A). To analyze the thermostability of FAST-PETase with or without the fusion tag, the target enzymes were incubated at 4°C, 28°C, 37°C, 50°C, and 55°C for 1 h, followed by an evaluation of their remaining activities at the optimal temperature. After incubation at 50°C for 1 h, the fused proteins remained almost full activities while less than 50% of the activity remained for FAST-PETase. After 1 h incubation at 55°C, FAST-PETase retained less than 40% of its activity while the fusion enzymes remained approximately 80% of their activities (Figure 4B).

### 3.4. Enzyme Kinetic of FAST-PETase Fused with or without S1v1 Tag

The kinetic parameters of FAST-PETase with or without S1v1 tag were investigated with *p*-NPB, which is a soluble substrate for cutinase. The binding strength (*K_M_*) and reaction speed (*k_cat_*) of FAST-PETase and S1v1-FAST-PETase with *p*-NPB were found to be similar (Table 1). However, FAST-PETase-S1v1 showed a much higher catalytic turnover, increasing by about 87.3% compared to FAST-PETase and 133.4% compared to S1v1-FAST-PETase. On the contrary, the affinity of FAST-PETase-S1v1 to *p*-NPB decreased by approximately 38.6% and 41.9% in comparison with FAST-PETase and S1v1-FAST-PETase, leading to approximately 35.2% and 64.1% increases in *k_cat_/K_M_* in comparison with FAST-PETase and S1v1-FAST-PETase, respectively (Table 1, Supplementary Figure S4). It is worth noting that the chemical characteristics of *p*-NPB are very different from those of insoluble PET. Therefore, the kinetic parameters were more likely to represent the cutinase activity of the enzymes, indicating FAST-PETase’s ability to break ester bonds.

### 3.5. Structure Simulation of the Interactions Between Enzymes and the Substrate

**Figure 5:**
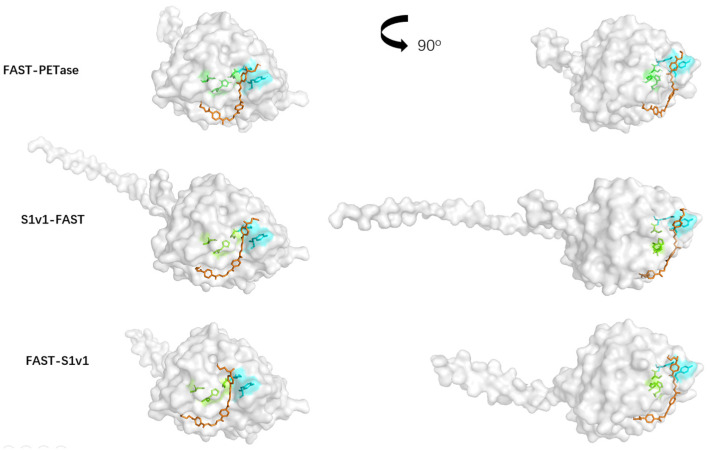
Molecular docking map of S1v1-tagged FAST-PETase with 3PET as the substrate. The Ser-His-Asp triad is labeled green. Tyr and Met in the oxyanion hole are labeled cyan. The substrate is labeled orange.

The structures of S1v1-FAST-PETase and FAST-PETase-S1v1 were predicted using AlphaFold3 (Supplementary Figure S5). The results indicated that FAST-PETase maintained the same structure regardless of the fusion of S1v1. However, the S1v1 multifunctional peptide demonstrated very different conformations when fused to the N- and C-terminus. N-terminal S1v1 was a random coil, while the C-terminal S1v1 was an α-helix. Subsequent molecular docking of the enzyme molecules was carried out, using 3PET as the substrate (Figure 5).

**Figure 6:**
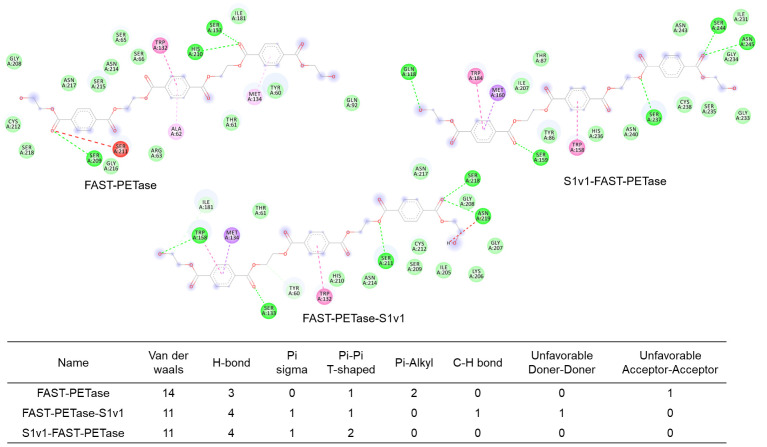
Calculation of non-covalent bonds formed between the enzymes and 3PET substrate.

The non-covalent bonds were calculated with Discovery Studio 2019 Client software. The results indicated the functional peptide increased hydrogen bonding between the enzymes and the substrate, while it decreased van der Waals forces (Figure 6), which indicated the enhanced catalytic performance was mainly caused by improved interactions between the substrate and the enzyme.

## 4. Discussions

Epitope tags, such as Maltose-binding protein (MBP), N-utilization substance A (NusA), small ubiquitin-modifying protein (SUMO), and glutathione S-transferase (GST), are widely utilized to enhance the folding of target proteins in the *E. coli* expression system, hence increasing the solubility of target proteins [31,32]. However, there is no universal standard for the selection of suitable fusion tags since the general mechanism remains unknown. Therefore, mining novel tags for high-efficiency heterologous expression remains a research hotspot. A previous report indicated that the CBM66 motif derived from *Bacillus subtilis* was able to promote the soluble protein expression and total yield of PETase [18]. Our results in the present study indicated that the S1v1 multifunctional peptide enhanced the properties of FAST-PETase. However, the sequences and amino acid composition of these two tags are very different. Moreover, the S1v1 tag demonstrated a very different structure when fused to N- and C-terminus of FAST-PETase. Therefore, it is difficult to analyze the effect of this multifunctional peptide on the enzyme. We deduced that S1v1 increased the interaction of the enzyme with the hydrophobic surface of the PET. In the future, more accurate structure simulation may provide more information to guide the design of the multifunctional epitope tags.

## 5. Conclusions

In summary, we developed an effective method for improving the expression and performance of FAST-PETase by fusing it with self-assembling amphipathic peptide S1v1. This approach enhances the solubility of FAST-PETase, boosting its catalytic activity and thermostability, and supports its use in PET degradation. In conclusion, the findings of this study establish the S1v1 fusion as a valuable tool for advancing enzymatic PET recycling, with potential for further optimization to enhance the production of other industrially useful enzymes.

## Figures and Tables

**Table 1:** Kinetics of FAST-PETase fused with S1v1 tag for *p*-NPB.

Enzyme	*k_cat_* (min^−1^)	*K_M_* (mmol·L^−1^)	*k_ca_*_t_/*K_M_* (mmol^−1^·L·min^−1^)
FAST-PETase	43.42 ± 3.19	0.88 ± 0.16	49.42 ± 6.70
S1v1-FAST-PETase	34.84 ± 2.35	0.86 ± 0.15	40.71 ± 4.33
FAST-PETase-S1v1	81.32 ± 5.51	1.22 ± 0.18	66.82 ± 8.25

Note: The experiment was performed in triplicate, and standard deviations are indicated.

## Data Availability

The original contributions presented in this study are included in the article/Supplementary Material. Further inquiries can be directed to the corresponding author.

## Supplementary Materials

The following supporting information can be downloaded at: https://www.mdpi.com/article/10.3390/biom15060888/s1, Figure S1: The amino acid sequence of S1v1-FAST-PETase; Figure S2: The amino acid sequence of FAST-PETase-S1v1; Figure S3: SDS-PAGE analysis of the purified FAST-PETase, S1v1-FAST-PETase and FAST-PETase-S1v1; Figure S4: Kinetics curves for enzymatic activities of FAST-PETase, S1v1-FAST-PETase and FAST-PETase-S1v1 with *p*-NPB as the substrate. The names of the enzymes are indicated at the top of the curves. Figure S5: The structures of S1v1-FAST-PETase and FAST-PETase-S1v1. A. The structural alignment of FAST-PETase (green) with S1v1-FAST-PETase (cyan); B. The structural alignment of FAST-PETase (green) and FAST-PETase-S1v1 (red). Figure S6: Uncropped and unedited western-blot images.
