# Supplementary material for: Improving the Catalytic Activity and Thermostability of FAST-PETase with a Multifunctional Short Peptide
Source: Biomolecules. 2025 Jun 18;15(6):888. doi: 10.3390/biom15060888 (PMC12190498; doi:10.3390/biom15060888)
Supplement: Supplementary file 1 [file biomolecules-15-00888-s001.zip › biomolecules-3686303-supplementary.pdf]

**Supplementary material**

```
        10         20         30         40         50         60
MAEAEAHAHA EAEAHAHTTV TTPQTSQTNP YARGPNPTAA SLEASAGPFT VRSFTVSRPS
        70         80         90        100        110        120
GYGAGTVYYP TNAGGTVGAI AIVPGYTARQ SSIKWWGPRL ASHGFVVITI DTNSTLDQPE
       130        140        150        160        170        180
SRSSQQMAAL RQVASLNGTS SSPIYGKVDT ARMGVMGWSM GGGGSLISAA NNPSLKAAAP
       190        200        210        220        230        240
QAPWHSSTNF SSVTVPTLIF ACENDSIAPV NSSALPIYDS MSQNAKQFLE IKGGSHSCAN
       250        260        270        280        290
SGNSNQALIG KKGVAWMKRF MDNDTRYSTF ACENPNSTAV SDFRTANCSH HHHHH
```

**Supplementary Figure S1. The amino acid sequence of S1v1-FAST-PETase.** The S1v1 tag is labeled in blue. PT-linker is labeled in green and 6×His tag is labeled in red.

.

```
        10         20         30         40         50         60
MQTNPYARGP NPTAASLEAS AGPFTVRSFT VSRPSGYGAG TVYYPTNAGG TVGAIAIVPG
        70         80         90        100        110        120
YTARQSSIKW WGPRLASHGF VVITIDTNST LDQPESRSSQ QMAALRQVAS LNGTSSSPIY
       130        140        150        160        170        180
GKVDTARMGV MGWSMGGGGS LISAANNPSL KAAAPQAPWH SSTNFSSVTV PTLIFACEND
       190        200        210        220        230        240
SIAPVNSSAL PIYDSMSQNA KQFLEIKGGS HSCANSGNSN QALIGKKGVA WMKRFMDNDT
       250        260        270        280        290
RYSTFACENP NSTAVSDFRT ANCSTTVTTP QTSAEAEAHA HAEAEAHAHH HHHHH
```

**Supplementary Figure S2. The amino acid sequence of FAST-PETase-S1v1.** The S1v1 tag is labeled in blue. PT-linker is labeled in green and 6×His tag is labeled in red.

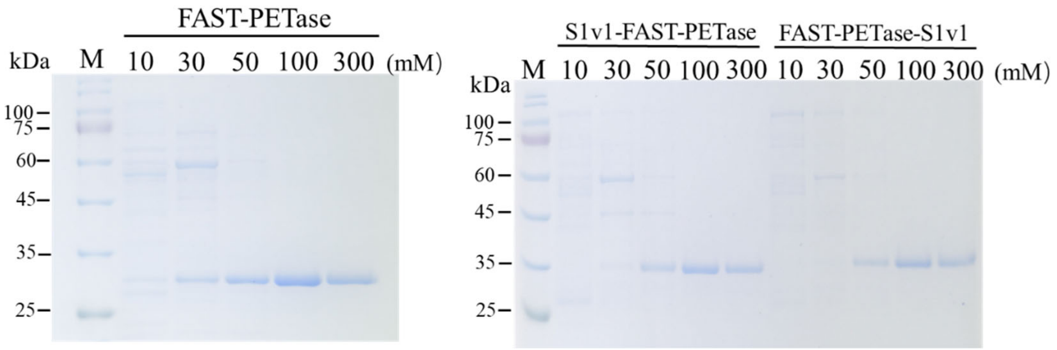


**Supplementary Figure S3. SDS-PAGE analysis of the purified FAST-PETase, S1v1-FAST-PETase and FAST-PETase-S1v1.**

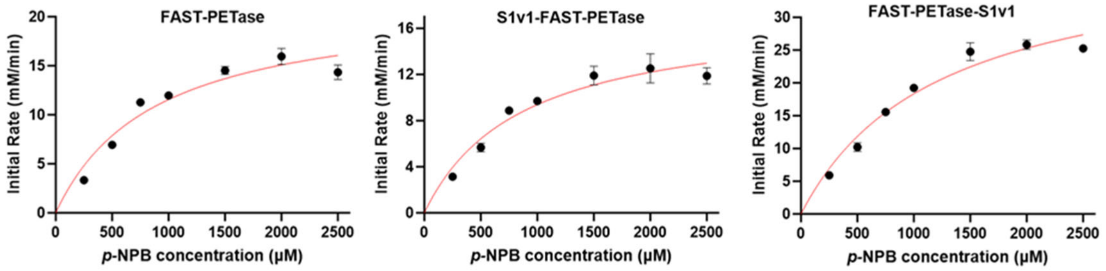


**Supplementary Figure S4. Kinetics curves for enzymatic activities of FAST-PETase, S1v1-FAST-PETase and FAST-PETase-S1v1 with *p*-NPB as the substrate.** The names of the enzymes are indicated at the top of the curves.

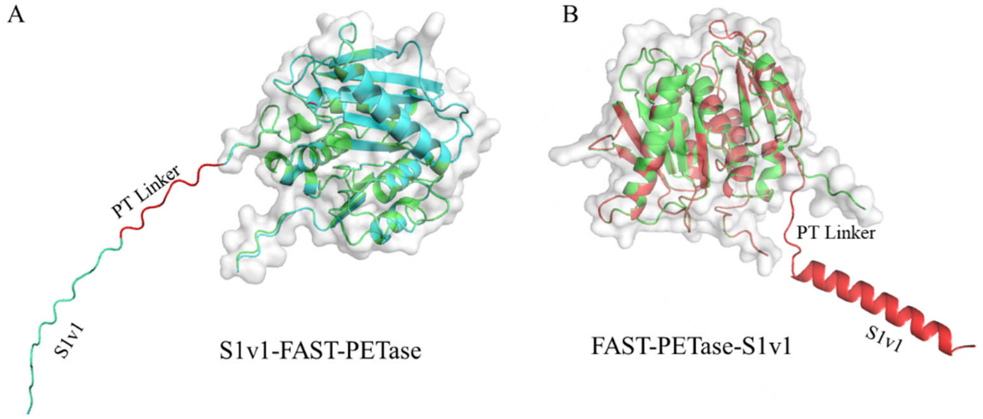


**Supplementary Figure S5. The structures of S1v1-FAST-PETase and FAST-PETase-S1v1.** A. The structural alignment of FAST-PETase (green) with S1v1-FAST-PETase (cyan); B. The structural alignment of FAST-PETase (green) and FAST-PETase-S1v1 (red).

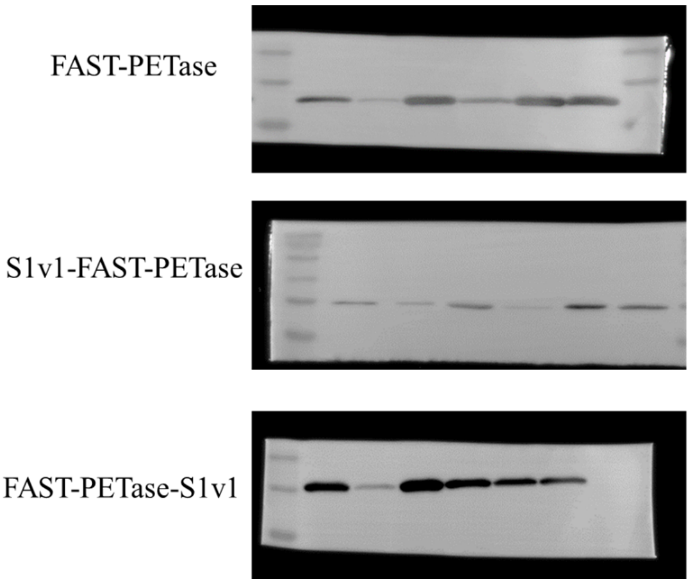


**Supplementary Figure S6. Uncropped and unedited western-blot images.**
